# Several components of postural control are affected by benign paroxysmal positional vertigo but improve after particle-repositioning maneuvers: A systematic review and meta-analysis

**DOI:** 10.1177/02692155241292662

**Published:** 2024-11-05

**Authors:** Sara Pauwels, Laura Casters, Pieter Meyns, Nele Lemkens, Winde Lemmens, Kenneth Meijer, Raymond van de Berg, Joke Spildooren

**Affiliations:** 1 Faculty of Rehabilitation Sciences, REVAL-Rehabilitation Research Centre, 54496 Hasselt University, Diepenbeek, Belgium; 2 Department of Otorhinolaryngology and Head & Neck Surgery, School for Mental Health and Neuroscience, Faculty of Health Medicine and Life Sciences, 199236 Maastricht University Medical Centre+, Maastricht, The Netherlands; 3 Department of Otorhinolaryngology, Head and Neck Surgery ZOL Hospital, Genk, Belgium; 4 Department of Nutrition and Movement Sciences, School of Nutrition and Translational Research in Metabolism, Faculty of Health Medicine and Life Sciences, Maastricht University, Maastricht, the Netherlands

**Keywords:** Postural control, benign paroxysmal positional vertigo, repositioning maneuvers, falls, vestibular disorders

## Abstract

**Objective:**

Benign Paroxysmal Positional Vertigo is a vestibular disorder causing vertigo and imbalance. This systematic review and meta-analysis aims to explore the impact of benign paroxysmal positioning vertigo and repositioning maneuvers on postural control.

**Data Sources:**

In September 2024, PubMed, Web of Science, Scopus and reference lists of included studies were systematically searched. Articles comparing measures of postural control between patients and controls, and/or pre- and posttreatment were considered relevant.

**Methods:**

Study selection, data extraction and identification of risk of bias were done by two researchers. If possible, meta-analysis was performed with Review Manager version 5.4.1 and standardized mean differences were calculated with a random-effects model.

**Results:**

Twenty-one of the 37 included studies were useful for meta-analyses. Meta-analyses revealed that benign paroxysmal positional vertigo negatively affects perception of verticality (p < .001; SMD = 0.73; 95% CI = [0.39;1.08]) and sensory orientation (p < .001; SMD = −1.66; 95% CI = [−2.08, −1.23]). The perception of verticality (p < .001; SMD = 0.99; 95% CI = [0.76;1.21]) and sensory orientation (p < .001; SMD = −0.77; 95% CI = [−1.11, −0.44]) improved after treatment with repositioning maneuvers. Results of systematic review indicate stability in gait was impaired, vertigo but improve after repositioning maneuvers. Limits of stability were impaired in older patients, but did not improved after repositioning maneuvers.

**Conclusion:**

Benign paroxysmal positioning vertigo affects several underlying components of postural control. Repositioning maneuvers can significantly improve the related postural control impairments. This may partly explain the increased odds of falling in these patients, and the positive treatment effect of repositioning maneuvers on falls and fear of falling.

## Introduction

Benign paroxysmal positional vertigo is a common peripheral vestibular disorder, diagnosed in 17%–42% of people with a complaint of vertigo, and a sevenfold higher prevalence in people over 60 years old (3.4%), compared to people under 40 (0.5%).^1,2^ It is caused by dislodged otoconia from the utricular macula that migrate into the semicircular canals. Typically, symptoms of vertigo and nystagmus are provoked when the head is moved in the plane of the affected semicircular canal.^3^ Although considered a benign disorder, people with benign paroxysmal positional vertigo, further referred to as patients, can experience a severe impact on quality of life,^4^ an increased odds of falling and altered spatiotemporal parameters of gait in comparison to their peers.^5^ The gold-standard treatment for benign paroxysmal positional vertigo are repositioning maneuvers, which involve a series of movements that aim to relocate the dislodged otoconia. It is well established that it can resolve signs and symptoms during positional testing, improve spatiotemporal parameters of gait, incidence, and fear of falling.^5,6^

**Figure 1.**
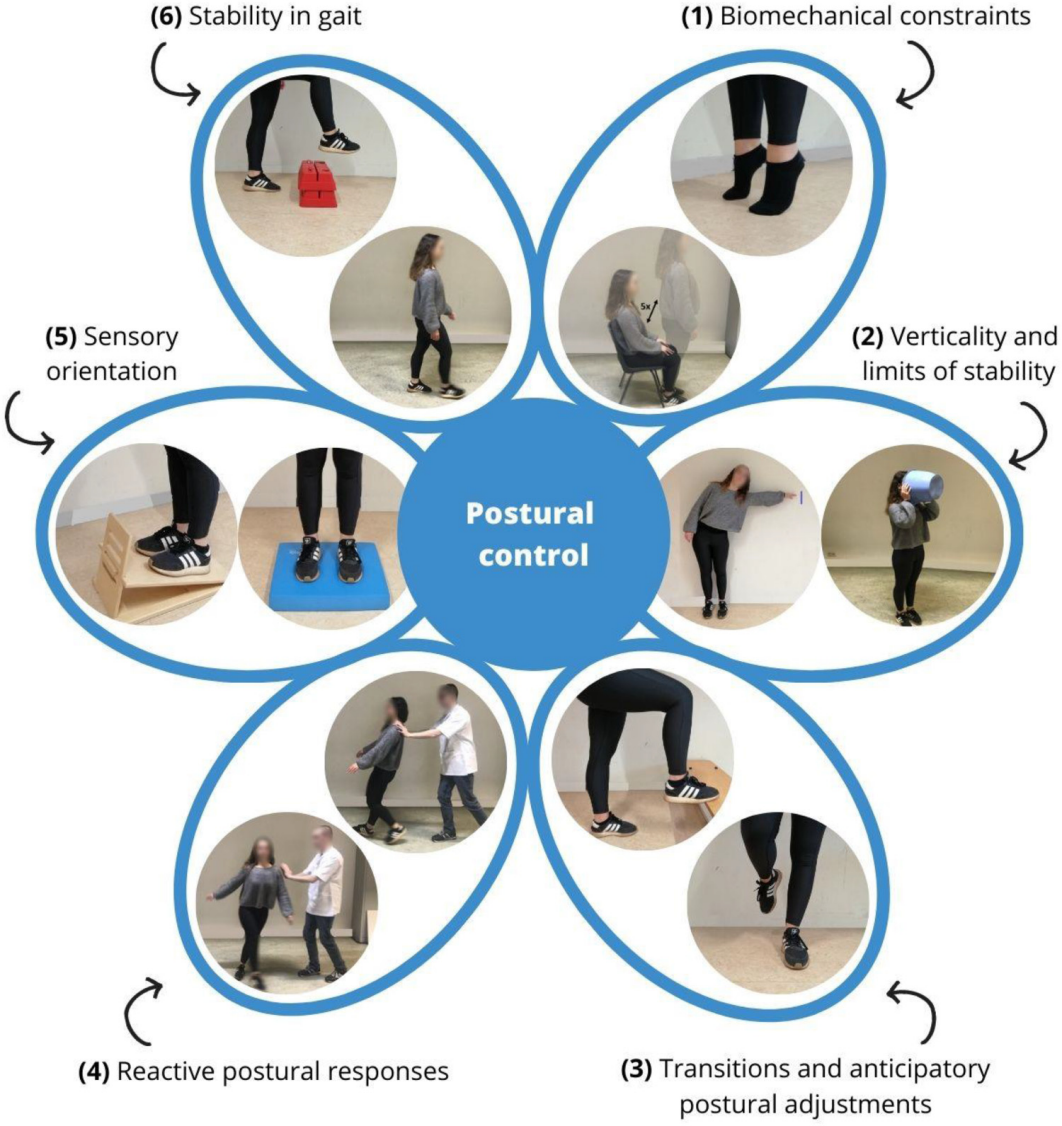
Systems framework for postural control.

Postural control, defined as maintaining or regaining the center of mass within the base of support, is a crucial function in the prevention of falls.^7^ It involves active control of body alignment with respect to gravity and support surface, and the coordination of sensorimotor strategies to stabilize the body's center of mass during internal and external perturbations. According to the systems framework, six components contribute to the maintenance of postural control: biomechanical constraints, verticality and limits of stability, transitions and anticipatory postural adjustments, reactive postural responses, sensory orientation, stability in gait*.*^7^ This framework has been proven to correlate with fear of falling and is able to discriminate between fallers and non-fallers.^7,8^ Definitions and examples of these components are provided in Figure 1.

Although it is known that patients have an increased odds of falling, comprehensive insights into which components of postural control are affected by benign paroxysmal positional vertigo or improve after repositioning maneuvers, are still lacking in the literature. Therefore, this systematic review aims to investigate the impact of benign paroxysmal positional vertigo and repositioning maneuvers on the different components of postural control.

## Methods

This study was conducted according to the preferred reporting items for systematic reviews and meta-analysis protocol (PRISMA).^9^ The protocol is available online at PROSPERO (www.crd.york.ac.uk/prospero; registration no. CRD42021261848).

In September 2024, a systematic literature search was performed by two independent reviewers (SP and LC), using the electronic databases PubMed, Web of Science and Scopus. To ensure no relevant articles were missed, references of included articles were also screened. Search strategies were based on synonyms for the keywords “benign paroxysmal positional vertigo” and “postural control” (more details in Supplementary Materials 1). No filters were applied.

Articles written in English, Dutch or French with a cohort, case-control or controlled study design were considered relevant. To be included, measures of postural control of adults with benign paroxysmal positional vertigo (≥18 years old) needed to be compared to those of controls. Articles comparing postural control pre- and post-treatment with repositioning maneuvers were included to measure the impact of repositioning maneuvers. Exclusion criteria were: (1) the presence of benign paroxysmal positional vertigo in combination with other disorders (e.g. Parkinson's disease) that could interfere with the outcome measures, (2) self-evaluation of postural control, (3) the use of (or combination of repositioning maneuver with) other treatments (e.g. vestibular rehabilitation), and (4) conference proceedings/reports, editorials, letters, case studies/series, (systematic) reviews and meta-analyses. Authors were contacted by email in case of unclarities. In case of multiple publications of the same subject sample and outcome measure, only the article with the largest sample size was retrieved for inclusion to avoid overrepresentation of these subjects.

Risk of bias was identified with the Joanna Briggs Institute critical-appraisal tools.^10^ The checklist for case-control studies was applied when outcome measures of benign paroxysmal positional vertigo were compared to controls. When patients received treatment with repositioning maneuvers, the checklist for quasi-experimental studies was used. Both checklists assessed the internal validity and the overall quality of the study. Articles were graded as “low risk of bias” (≥70% yes-score), “moderate risk of bias” (50–69% yes-score) or “high risk of bias” (<49% yes-score).^11^ Studies with a high risk of bias were excluded.

All studies were assessed by two independent researchers (SP and LC). The rating method was standardized and results were discussed in a consensus meeting. If consensus was not reached, a third researcher (JS) was consulted.

General population characteristics (number of participants per group, mean(SD) age, age range, sex distribution), specific characteristics of patient groups, treatment (affected semicircular canal, repositioning maneuver, number of treatment sessions, follow-up after treatment), and how patients were screened for coexisting vestibular disorders were collected.

Results on postural control were classified according to the specific component tested: “biomechanical constraints”, “verticality”, “limits of stability”, “transitions and anticipatory postural control”, “reactive postural control”, “ sensory orientation” and “stability in gait”. Since spatiotemporal parameters of gait were described in a previous systematic review,^5^ they were not included in this paper. Total results on the Berg Balance Scale were reported as “generic balance”. If multiple measurements posttreatment were reported, data from the earliest measurement were derived for the synthesis. Tests and outcome measures used for the components of postural control are provided in Supplementary Materials 2.

Numeric values (mean and SD) for each outcome were extracted. When median and range were reported, mean variance and standard deviation were estimated by the method of Hozo et al.^12^ If an outcome measure was discussed in 3 articles or more,^13^ a meta-analysis of the raw data was executed with Review Manager version 5.4.1. To conduct the meta-analysis, the mean, standard deviation and number of participants in each group were used. Standardized mean differences (SMD) were calculated with a random-effects model for continuous variables.

Confidence intervals were set at 95%. A significance level of p < 0.05 was applied to all outcome measures. For sensory orientation, outcome measures of center of pressure and center of gravity were grouped as “sway area” (path length (mm), area (cm^2^), stillness (%)), “sway velocity” (velocity (°/s or cm/s), end-sway velocity (°/s), peak velocity (cm/s)) and “accelerations” (range (cm/s^2^), root mean square). Equilibrium scores and performance time were also reported.

Heterogeneity between the publications was measured by the Higgins I² statistic^14^ and was classified as low (<50%) moderate (<75%) or high (>75%). Only moderate and high heterogeneity were described in the text. When no raw data was available in the article, the authors of the corresponding article were contacted by email. Outcomes that could not be included in a meta-analysis were described.

## Results

### Literature search

**Figure 2.**
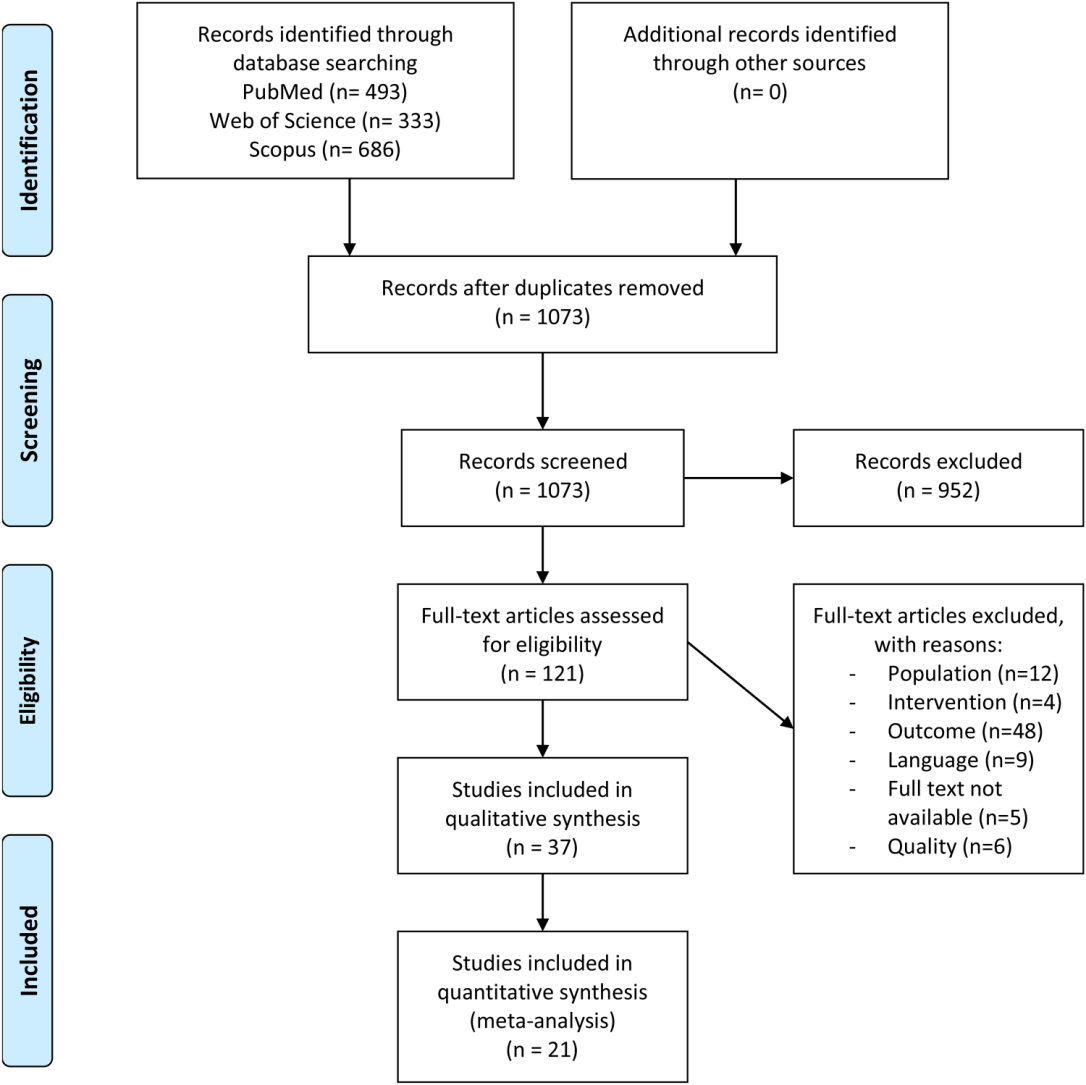
PRISMA 2020 flow diagram.^9^

In September 2024, a systematic literature search was conducted on PubMed, Web of Science and Scopus (Figure 2).^9^ The reference lists were also screened for potentially relevant articles. The search query revealed 1073 unique citations. Thirty-seven of the 121 studies that were assessed for eligibility were included in the review. Twenty-one studies were included in the meta-analysis, the remaining 16 studies were used for descriptive data only. The number of included studies for the different components of postural control varied, and are therefore presented in Figure 2.

### Risk of bias in individual studies

Seventeen studies comparing postural control between patients and controls were assessed with the Joanna Briggs Institute critical-appraisal checklist for case-control studies.^15–31^ Five studies were classified as high risk of bias^15,19–21,27^ and therefore excluded. Four studies were classified as moderate risk^18,22,23,29^ and eight as low risk of bias.^16,17,24–26,28,30,31^ Appropriate matching between patients and controls was done in four studies.^18,25,26,28^ In four studies, the presence of nystagmus was checked with the use of defocusing goggles (e.g. frenzel, videonystagmography), which is believed to improve diagnostic accuracy.^28–31^ In four studies, the presence of benign paroxysmal positional vertigo was not checked with diagnostic maneuvers in the control group.^18,23,25,29^

Twenty-six studies comparing postural control pre- and post-treatment with repositioning maneuvers were assessed with the Joanna Briggs Institute critical-appraisal checklist for quasi-experimental studies.^32–57^ One study was classified as high risk of bias and was therefore excluded.^49^ Ten studies were classified as moderate risk^35,37,38,40,42,50,52,53,55,56^ and fifteen as low risk of bias.^32–34,36,39,41,43–48,51,54,57^ Eight studies had a single-group pre-test/post-test design.^35,38,40,42,52,53,55,56^ Therefore, differences in treatment/care or ways to measure the outcome between groups were not applicable in these studies. There was no attrition bias, since follow-up was completed in all 25 studies. Power calculations were performed in six studies.^43–46,48,57^ Reliability of measurements was sufficient in seven studies.^32,36,39,43,44,46,57^

An overview of the risk-of-bias assessment for case-control and quasi-experimental studies can be found in Supplementary Material 3.

### Study and population characteristics

In total, 1208 patients and 1241 controls were included with a mean age from 42.80^36^ to 79^31^ years old and from 34.5^17^ to 78^31^ years old, respectively. In 24 studies,^16–18,22,23,25,29,30,32–35,37,39,41–43,46–48,51,53,55,57^ only posterior-canal benign paroxysmal positional vertigo was included, while 12 studies also included lateral- and/or anterior-canal benign paroxysmal positional vertigo.^23,26,28,31,36,38,44,45,50,52,54,56^ In one study, the affected canal was not specified.^40^ Nine studies performed vestibular function tests to exclude patients with a coexisting vestibular disorder.^24,30,32,36,45,48,52,55,56^ Thirteen studies screened medical history only.^23,25,28,33,38,41,43,44,46,48,50,54^ In three studies, vestibular function tests revealed peripheral changes, but these patients remained included.^26,31,47^ Twelve studies did not report any use of vestibular function tests or screening of the medical history for coexisting vestibular disorders.^16–18,22,34,35,39,40,42,51,53,57^ Nine hundred eighteen patients received treatment with repositioning maneuvers. Posterior-canal benign paroxysmal positional vertigo was treated with the Epley,^22,33,34,37–39,41–44,46,48,50–57^ modified Epley,^32,35,44,45^ augmented Epley,^56^ self-Epley,^44^ Semont^49,53^ or Gans maneuver.^34^ Involvement of the lateral canal was treated with the barbeque roll^45,52,56^ or Gufoni maneuver.^50,52^ The Epley^54^ maneuver was applied to treat anterior-canal involvement. In two studies, the repositioning maneuver was not specified.^36,40^ Timing of the first measurement post-treatment ranged from immediately after repositioning maneuver^37,38^ to two weeks after repositioning maneuver.^32^ An overview of study and population characteristics is provided in Table 1.

**Table 1.** Study and population characteristics.

Study		BPPV			Treatment			Coexisting Vestibular disorders	Control	
Author	Design	N (F/M)	Affected canal	Age in years (Mean ± SD)	Repositioning maneuver		Follow-up		N (F/M)	Age in years (Mean ± SD)
Abou-Elew et al.^56^	Prospective	19 (11/8)	PC: 16 LC: 3	47.42 ± 9.2	Epley BBQ		1 week after successful RM	History ENG	Age-matched but not further specified	
Agarwal et al.^16^	Case-control	20 (16/4)	PC	60 ± 13.7				/	20 (16/4)	54.8 ± 11.6
Assal et al.^36^	Prospective case-control	20 (15/5)	PC: 15 LC: 4 AC: 1	42.80 ± 9.71	Not specified		1 week	Caloric test History	20 (15/5)	44.75 ± 9.51
Best et al.^30^	Prospective	11 (6/5)	PC	56.00 ± 11.39				ENG	26 (13/13)	33 ± 13.35
Blatt et al.^55^	Prospective	33 (26/7)	PC	57.4 ± 17.9	Liberatory		1−2 weeks	History ENG		
Bulğurcu et al.^57^	Prospective	48 (38/10)	PC	48.5 ± 12.63	Epley		1 week	/		
Çelebisoy et al.^45^	Prospective	44	PC: 32 LC: 12	55 (range: 32–77) 55.6 (range: 39–74)	Modified Epley BBQ		1 week 2 weeks	History Caloric test	50	48.3 (range 27–70)
Chang et al.^32^	RCT	13 (7/6)	PC	53.93 ± 9.97	Modified Epley		2 weeks 4 weeks	History ENG		
Cohen & Kimball^51^	RCT	76 (53/23)	PC	56 (range 27–81)	Epley Augmented Epley Epley + home instructions		1 week 3 months 6 months	/		
Cohen & Sangi- Haghpeykar^44^	RCT	92 (90/2)	PC: 79 PC + LC: 13	56.9 ± 12.9	Epley Modified Epley Self-Epley		1 week 3 months 6 months	History		
Cohen and Sangi- Haghpeykar^29^	Prospective case-controlled	25 (19/6)	PC: 79	60.1 ± 13.2				History	50 (31/19)	49.5 ± 11
Cohen et al.^23^	Prospective	21 (11/10)	PC + LC: 13	58.8 ± 11.7				History	61 (30/31)	49.6 ± 16.0
Cohen et al.^24^	Prospective	21 (11/10)	PC	58.8 ± 11.7				VNG	156 (76/80)	52.8 ± 18.0
Cohen-Shwartz et al.^46^	Prospective	32 (25/7)	PC	64.3 ± 6.4	Epley		1 week	History	15 (9/6)	63.5 ± 7.1
D'Silva et al.^25^	Prospective	13 (11/2)	PC	54.5 ± 6.0				History of menière	14 (11/3)	58.07 ± 4.9
Di Girolamo et al.^47^	Prospective	32 (25/7)	PC	51.9	Semont		3 days 1 month	28% had assymetrical caloric test	32	gender- and age matched
Faralli et al.^48^	Prospective cohort	116 (74/42)	PC	54.3 ± 7.9	Epley		1 week	History	40	gender- and age matched
Kasse et al.^54^	Prospective	33 (29/4)	PC: 27 Bi. PC: 5 AC: 1	68.39 ± 5.52	Epley		2 weeks	History	33 (12/21)	69.97 ± 4.48
Kollén et al.^53^	Prospective	17 (13/4)	PC	52 (range: 31–66)	Semont		1 month 6 months 12 months	/		
Kollén et al.^22^	Cross-sectional	63 (46/17)	PC	75				/	508 (286/222)	75
Lança et al.^40^	Prospective	21 (17/4)	Not specified	68.74	Not specified		After PRM 12 months	/		
Lee et al.^28^	Case controlled	49 (27/22)	PC: 18 LC: 31	51.2 ± 12.6				History	30 (14/16)	45.6 ± 12.8
Lim et al.^52^	Prospective	33 (23/11)	PC: 24 LC: 10	60.20 ± 11.94		Epley BBQ Gufoni	mean: 8.73 day (SD: 5.94)	VNG vHIT		
Lindell et al.^31^	Prospective	15 (14/1)	PC: 10 LC: 5	79 ± 3.8				Pathological vHIT in 13.3%	40 (38/2)	78 ± 4.5
Faralli et al.^37^	Prospective	30 (17/13)	PC	52 ± 9.4		Epley	After RM 7 days	History Caloric test, head- shaking test & mastoid oscillation	20	Not specified
Ferreira, Ganança, and Caovilla^38^	Prospective	20 (16/4)	PC: 19 LC: 1	58.35 (range 51–89)		Epley BBQ	After RM	History		
Monteiro et al.^26^	Prospective case-controlled	45 (33/12)	PC: 35 Bi. PC: 5 AC: 3 LC: 1 LC + PC: 1	49.13 ± 9.53				46.6% had peripheral changes on VNG	45 (36/9)	45.62 ± 11.84
Mulavara et al.^17^	Prospective	26	PC	59.2 ± 11.6				/	14	34.5 ± 9.1
Navarro et al.^50^	Prospective	46 (28/18)	PC: 39 LC: 7	60.24 ± 16.52		Epley Gufoni	After resolution	History		
Omara et al.^34^	RCT	30 (18/12)	PC	51.9 ± 5.9		Epley Gans	After complete remission	/		
Ribeiro et al.^39^	RCT	7 (5/2)	PC	71.75 ± 3.15		Epley	1 week 5 weeks 9 weeks 13 weeks	/		
Silva et al.^42^	Prospective, quasi-experimental	14 (11/3)	PC	71 ± 4.05		Epley	1 week	/		
Stambolieva & Angov^33^	Prospective	20	PC	53.3 ± 8.4		Epley	7 days	History	20	50.1 ± 9.8
Stambolieva & Angov^41^	Prospective	47	PC	55.8 ± 5.5		Epley	One hour 10 days 20 days	History	20	54.2 ± 7.9
Taçalan et al.^43^	RCT	18	PC	46.11 ± 9.82		Epley	1 week after 3 weeks after 6 weeks after	History		
Vaz et al.^35^	Prospective clinical	30 (28/2)	PC	70.10 ± 7.00		Modified Epley	1 week	/		
Zhang et al.^18^	Prospective	27 (16/11)	PC	56.5 ± 13.1				/	27 (21/6)	56.1 ± 10.8

Abbreviations: BPPV, Benign Paroxysmal Positional Vertigo; N, number of participants; F, female; M, male; SD, standard deviation; RM, particle-repositioning maneuver; PC, posterior semicircular canal BPPV; LC, lateral semicircular canal BPPV; BBQ, Barbeque Roll maneuver; AC, anterior semicircular canal BPPV; Bi. PC: bilateral posterior semicircular canal BPPV; RCT, randomized controlled trail; ENG, electronystagmography; VNG, videonystagmogrpahy; vHIT, video Head Impulse Test.

### Results on postural control

**Figure 3.**
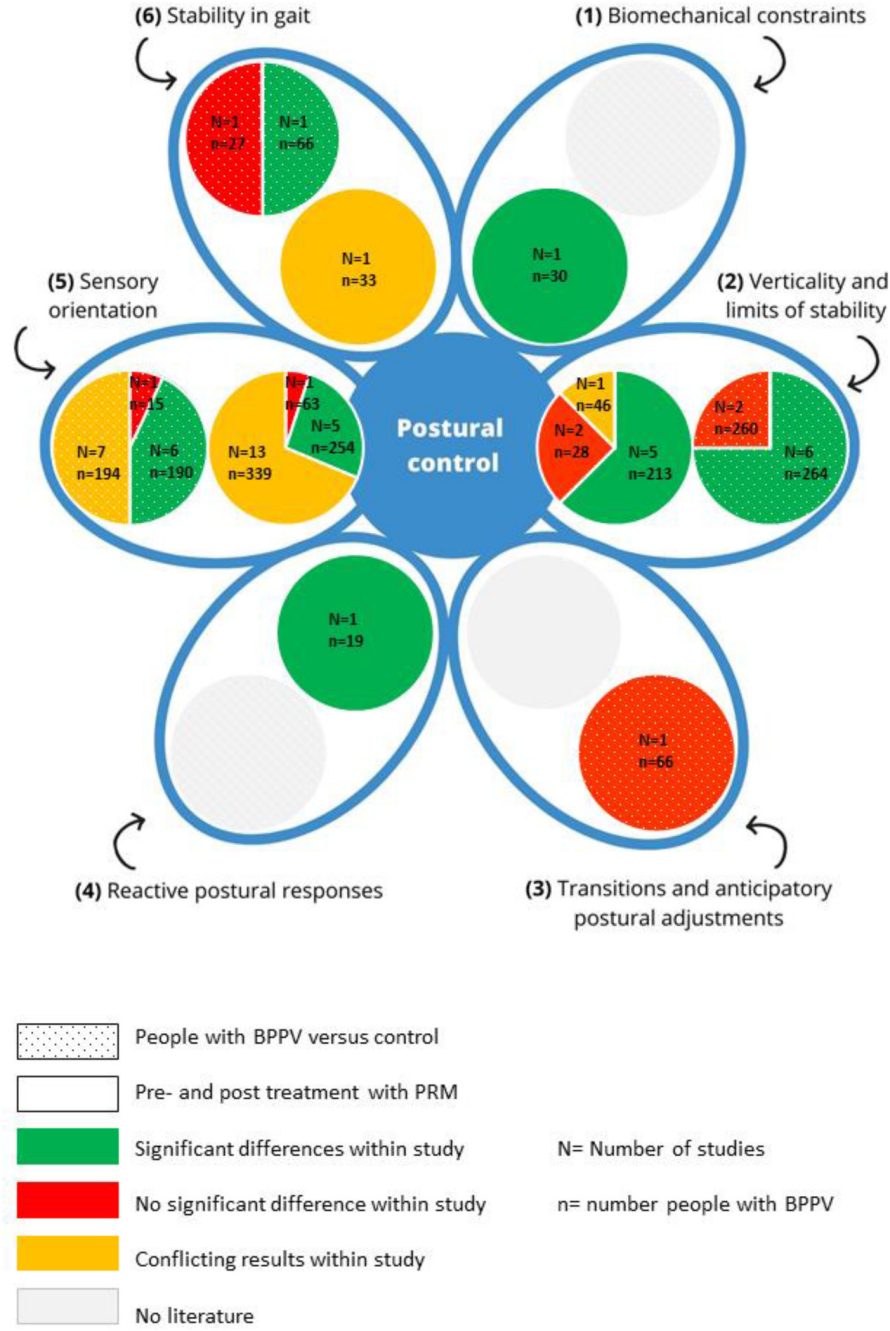
Summary of results on postural control.

A summary of the results and the number of included studies in each component of postural control is provided in Figure 3.

The *Berg Balance Scale (generic balance)* contains 14 items that require subjects to perform different tasks that vary from transfers to turning and tasks (e.g. looking over shoulders) while standing.

Differences between patients and controls were not reported in the included studies, but two studies^43,57^ reported a significant improvement of the total score after repositioning maneuvers.

Differences in *biomechanical constraints* between patients and controls were not reported in the included studies.

One study reported a significant improvement in performance time of the timed chair stand test in older adults after repositioning maneuver.^35^ During the timed chair stand test patients were asked to stand up from a chair five times, with their arms crossed on their chest.

The *perception of verticality* (Table 2) was compared between patients and controls with the subjective visual vertical in six studies*.*^28–31,37,48^ The subjective visual vertical was assessed with the bucket test^29,31^ and a light bar.^28,30,37,48^ In both tests, the participant was instructed to align a bar to the perceived earth vertical. Meta-analyses revealed a significantly increased deviation from the true vertical in patients, with moderate heterogeneity. Results of Best et al. could not be pooled as only p-values and graphs were available, but were in line with the meta-analyses.^30^ Lindell et al. found no significant difference in number of persons with an abnormal subjective visual vertical between patients and controls.^31^

**Table 2.** Results of meta-analyses on verticality.

	SMD; [95% CI]	P-value	I^2^	ref	S. Mat
pwBPPV versus control					4
Deviation from true vertical (°) (N = 4; pwBPPV = 220, control = 140)	0.73; [0.39;1.08]	**<0** **.** **001**	52%	^28,29,37,48^	4a
Treatment effect of repositioning maneuvers					
Deviation from true vertical (°) (N = 3; pwBPPV = 166)	0.99; [0.76;1.21]	**<0**.**001**	0%	^37,38,48^	4b

Abbreviations: SMD, standardized mean difference; N, number of pooled studies; pwBPPV, people with BPPV pooled for the included studies; COG, center of gravity; BoS, base of support; S. Mat., Supplementary Materials. Significant differences are indicated in bold.

Meta-analyses of three studies revealed a significant decrease in subjective visual vertical deviation after repositioning maneuvers.^37,38,48^

*Limits of stability* was compared between patients and controls in two studies. One study assessing limits of stability area in a group of older adults, reported a significantly smaller limits of stability area in patients.^54^ However, one study including a younger age group, did not confirm this difference.^26^

The impact of repositioning maneuvers on limits of stability area^40,54^ and limits of stability movement velocity and maximum excursion^39,42,50^ was assessed in four studies including older patients (≥60 years old),^39,40,42,54^ and one study comparing younger to older patients.^50^ Results on limits of stability area in older patients were conflicting.^40,54^ Two^39,50^ out three studies^39,42,50^ assessing movement velocity and maximum excursion found no improvement in older patients, whereas younger patients^50^ improved. Results could not be pooled due to missing standard deviations in Navarro et al.^50^

One study investigating *transitions and anticipatory postural control* found no significant difference between patients and controls, as assessed by the Functional Mobility test.^23^ In this obstacle-avoidance task, the number of obstacles touched and time to complete the test were measured.

None of the included studies reported on the impact of repositioning maneuvers.

Differences in *reactive postural responses* between patients and controls were not reported in the included studies.

One study reported a significant improvement on the motor control test after repositioning maneuvers in subjects with abnormal baseline scores.^56^ During the motor control test, a force platform is unexpectedly moved forward and backward, and the amount of sway during the response is measured.

**Figure 4.**
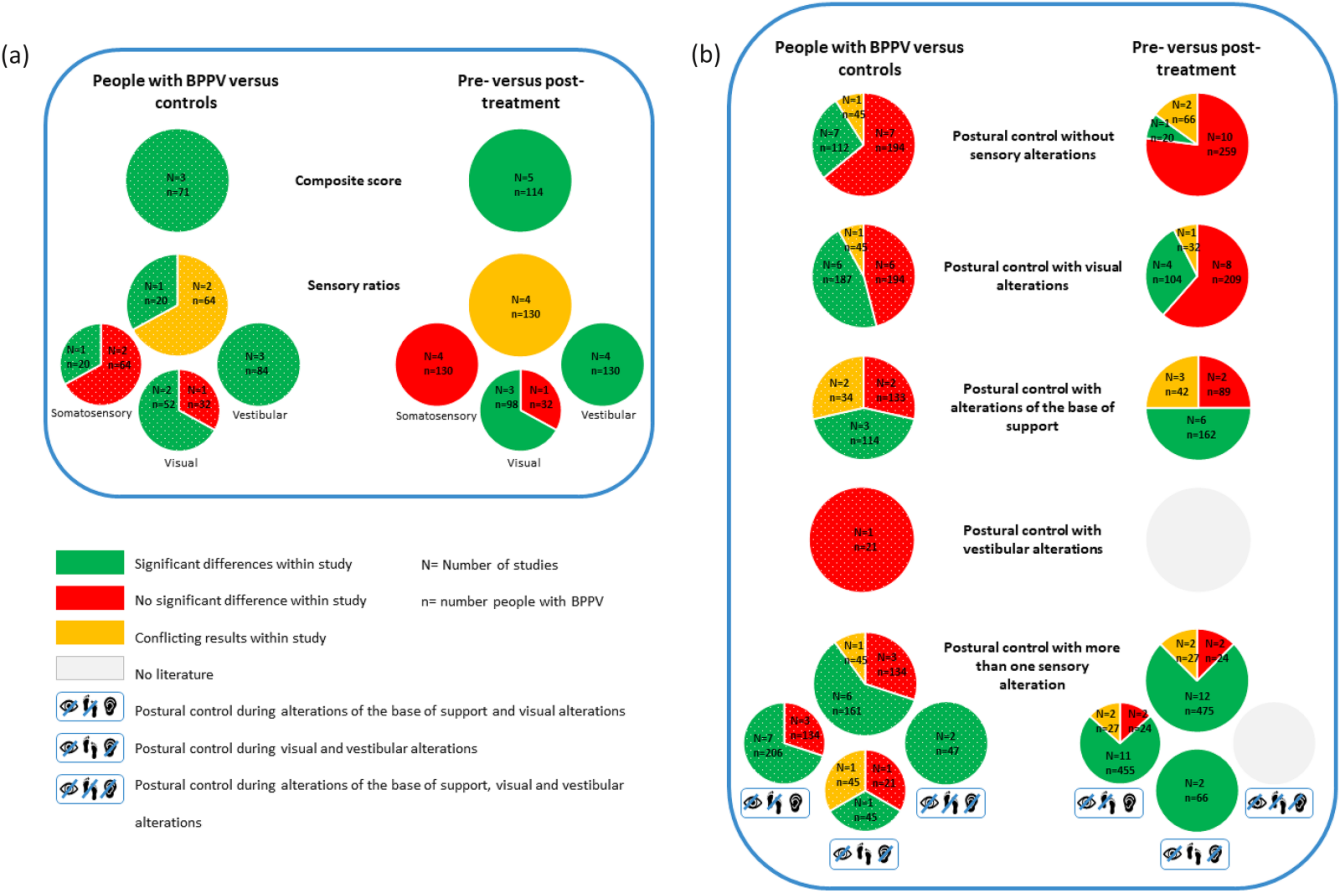
(a) Summary of results on composite scores and sensory ratios. (b) Summary of results on the conditions of sensory orientation.

In 14 studies, *sensory orientation* of patients was compared to controls.^16,17,24–26,31,33,36,41,45,46,49,54,56^ In 19 studies, the impact of repositioning maneuvers on sensory orientation was assessed.^32–36,39,40,42–47,50,51,53–56^ “Composite scores and sensory ratios” were discussed (Figure 4(a)). Next, conditions of sensory orientation were stratified according to the sensory alteration applied: “without sensory alterations”, “visual alterations”, “alterations of the base of support”, “vestibular alterations”, “more than one sensory alteration” (Figure 4(b)). Results of the meta-analyses on composite score and sensory ratio are summarized in Tables 3 and 4, respectively. Results of the meta-analyses of impact of repositioning maneuvers on sensory orientation are summarized in Table 5.

**Table 3.** Results of meta-analyses on composite score.

	SMD; [95% CI]	P-value	I^2^	ref	S. Mat
pwBPPV versus control					5
Average equilibrium score (N = 3, pwBPPV = 64)	−1.66; [−2.08, −1.23]	**<0** **.** **001**	8%	^36,47,56^	5a
Treatment effect of repositioning maneuvers					
Average equilibrium score (N = 4; pwBPPV = 104)	−0.71; [−1.00, −0.43]	**<0**.**001**	0%	^36,47,55,56^	5b

Abbreviations: SMD, standardized mean difference; N, number of pooled studies; pwBPPV, people with BPPV pooled for the included studies; COG, center of gravity; BoS, base of support; S. Mat., Supplementary Materials. Significant differences are indicated in bold.

**Table 4.** Meta-analyses of treatment effect of repositioning maneuvers on sensory ratios.

	SMD; [95% CI]	P-value	I^2^	ref	S. Mat
Vestibular ratio (N = 3, pwBPPV = 98)	−0.67 [−0.96, −0.39]	**<0** **.** **001**	0%	^36,47,50^	6a
Visual ratio (N = 3; pwBPPV = 98)	−0.43; [−0.72, −0.15]	**0**.**003**	0%	^36,47,50^	6b
Somatosensory ratio (N = 3; pwBPPV = 98)	−0.23; [−0.51, −0.05]	**0**.**11**	0%	^36,47,50^	6c

Abbreviations: SMD, standardized mean difference; N, number of pooled studies; pwBPPV, people with BPPV pooled for the included studies; S. Mat., Supplementary Materials. Significant differences are indicated in bold.

**Table 5.** Meta-analyses of treatment effect of repositioning maneuvers on sensory orientation.

	SMD; [95% CI]	P-value	I^2^	ref	S. Mat
Postural control without sensory alterations					7
Equilibrium score (N = 3; pwBPPV = 84)	−0.03; [−0.33, 0.28]	0.86	0%	^47,55,56^	7a
COG sway velocity (°/s) (N = 3; pwBPPV = 65)	−0.21; [−0.56, 0.13]	0.23	0%	^39,42,45^	7b
Postural control with visual alterations					8
*Eyes closed*					
Equilibrium score (N = 3; pwBPPV = 84)	−0.08; [−0.38, 0.23]	0.62	0%	^47,55,56^	8a
COG sway velocity (°/s) (N = 3; pwBPPV = 65)	0.08; [−0.34, 0.50]	0.73	19%	^39,42,45^	8b
*Altered visual information*					
Equilibrium score (N = 3; pwBPPV = 84)	−0.06; [−0.43, 0.31]	0.76	31%	^47,55,56^	8c
Postural control with alterations of the BoS					9
*Sway referenced support*					
Equilibrium score (N = 3; pwBPPV = 84)	−0.63; [−1.11, −0.15]	**0**.**01**	56%	^47,55,56^	9a
*Foam*					
COG sway velocity (°/s) (N = 4; pwBPPV = 78)	0.42; [−0.05, 0.89]	0.08	41%	^32,39,42,45^	9b
*One leg stance*					
COG sway velocity (°/s) (N = 3; pwBPPV = 34)	0.45; [−0.19, 1.10]	0.17	40%	^32,39,42^	9c
Postural control with more than one sensory alteration					10
*Sway-referenced support surface with eyes closed*					
Equilibrium score (N = 4; pwBPPV = 176)	−0.69; [−0.91, −0.47]	**<0**.**001**	0%	^44,47,55,56^	10a
*Sway-referenced support surface and visual surround*					
Equilibrium score (N = 3; pwBPPV = 84)	−0.69; [−1.00, −0.37]	**<0**.**001**	0%	^47,55,56^	10b
*Standing on a foam surface with eyes closed*					
COG sway velocity (°/s) (N = 4; pwBPPV = 78)	−0.45; [0.13, 0.77]	**0**.**006**	0%	^32,39,42,45^	10c
*One-leg stance with eyes closed*					
COG sway velocity (°/s) (N = 3; pwBPPV = 34)	−0.46; [−0.06, 0.99]	0.08	11%	^32,39,42^	10d

Abbreviations: SMD, standardized mean difference; N, number of pooled studies; pwBPPV, people with BPPV pooled for the included studies; COG, center of gravity; BoS, base of support S. Mat., Supplementary Materials. Significant differences are indicated in bold.

*Composite score* is a weighted average of the equilibrium scores of six conditions of the sensory organization test. Meta-analysis revealed a significantly decreased composite score in patients.^36,47,56^

Meta-analysis revealed a significant improvement in composite score after repositioning maneuvers.^36,47,50,55,56^ Due to missing data (i.e. standard deviations and overall score), composite scores of one study could not be pooled, but the results were in line with the meta-analysis.^34^

*Sensory ratios* indicate the contribution of the sensory systems during postural control and are a significant improvement of calculated with scores of the sensory organization test^36,47,50^ or clinical test of sensory interaction on balance.^46^ All studies reported a decreased vestibular ratio in patients.^36,46,47^ The visual ratio was decreased in two^36,47^ studies, while the somatosensory ratio was decreased in only one^36^ study.

Meta-analyses revealed a significant improvement after repositioning maneuvers in the vestibular and visual ratio, but not in the somatosensory ratio.^36,47,50^ Cohen-Shwartz et al. only reported improvement in the vestibular ratio. Their results were not pooled due to different measurement techniques.^46^

*Postural control without sensory alterations* was compared between patients and controls (i.e. standing on a firm surface, eyes open) in 11 studies.^16,24–26,33,41,45–47,54,56^ No significant differences were found in equilibrium score.^47,56^ Results on sway area^16,26,46,54^ and sway velocity^26,33,41,45,54^ were conflicting. No significant difference was found for accelerations^25^ or performance time.^24^

The impact of repositioning maneuvers was assessed in 13 studies.^33–35,39,40,42,43,45–47,54–56^ Meta-analysis of equilibrium scores and center of gravity sway velocity did not reveal a significant improvement after repositioning maneuvers. Results on equilibrium scores of Omara et al. were in line with the meta-analysis.^34^ Sway area^40,43,46^ did not change, while center of pressure sway velocity decreased.^33,40,54^ Performance time^35^ did not improve after repositioning maneuvers.

*Postural control with visual alterations* was compared between patients and controls in 13 studies (i.e. standing with eyes closed^16,22,24–26,31,33,41,45–47,54,56^ and altered visual input^26,47,54,56^). Equilibrium scores during eyes closed and altered visual input were significantly decreased in patients in the study with a larger sample size,^47^ but not in the study with a smaller sample size.^56^ During eyes closed, three^16,26,54^ out of four^16,26,46,54^ studies found an increased sway area, while three^26,33,41^ out of five^26,33,41,45,54^ studies found an increased sway velocity in patients. Accelerations were also increased,^25^ but no significant difference was found in performance time.^22,24,31^ During altered visual input, results on sway area and velocity were conflicting.^26,54^

The impact of repositioning maneuvers on postural control during visual alterations was assessed in 13 studies (i.e. standing with eyes closed^33–35,39,40,42,43,45–47,54–56^ and altered visual input^34,40,47,54–56).^ Meta-analysis of equilibrium scores and center of gravity sway velocity revealed no significant improvements during eyes closed. Results on equilibrium scores of Omara et al. were in line with the meta-analysis.^34^ During eyes closed, sway area decreased in three^40,43,54^ out of four^40,43,46,54^ studies, while all studies found a decreased center of pressure sway velocity.^33,40,54^ During altered visual input, results on sway area and velocity were conflicting.^40,54^ Performance time improved significantly during altered visual input^35^ but not during eyes closed.^35,53^

*Postural control with alterations of the base of support* was compared between patients and controls in seven studies (i.e. sway-referenced support,^47,56^ foam,^24,25,45,46^ one-leg stance^22^ and tandem stance^22,25^). Equilibrium scores were significantly decreased in patients.^47,56^ Sway area^46^ and velocity^45^ did not differ. Results on accelerations were conflicting.^24,25^ Performance time was significantly decreased when base of support was reduced (i.e. one-leg stance & tandem stance^22^ but not during bipedal stance on foam.^24^

The impact of repositioning maneuvers was assessed in 12 studies (i.e. sway-referenced support,^34,47,55,56^ foam^32,35,39,40,42,45,46^ and one-leg stance^32,39,42,43^). Meta-analysis of equilibrium scores revealed a significant improvement, with moderate heterogeneity. Results on equilibrium scores of Omara et al. were in line with the meta-analysis.^34^ However, meta-analysis of center of gravity sway velocity while standing on a foam surface and single-leg stance revealed no significant improvement after repositioning maneuvers. Accordingly, sway area did not improve.^40,43,46^ Center of pressure sway velocity^40^ and performance time for standing on foam significantly increased after repositioning maneuvers.^35^

*Postural control with vestibular alterations* was compared between patients and controls in one study.^24^ They reported no significant difference in performance time during head movements (pitch and yaw).

None of the included studies reported on the impact of repositioning maneuvers.

*Postural control with more than one sensory alteration* was compared between patients and controls in 10 studies.^17,22,24–26,45–47,54,56^ In 16 studies, the impact of repositioning maneuvers was assessed.^32,34,35,39,40,42–47,51,53–56^

Postural control during alterations of base of support (i.e. sway-referenced support,^47,56^ foam,^17,24–26,45,46,54^ tandem stance^22^) in combination with visual alterations (i.e. eyes closed and moving visual scene^47,56^) was compared between patients and controls in 10 studies. Equilibrium scores,^47,56^ sway area,^26,46,54^ sway velocity^26,45,54^ and accelerations^24,25^ were significantly decreased in patients. Results on performance time were conflicting.^17,22,24^

The impact of repositioning maneuvers on postural control during alterations of base of support (i.e. sway-referenced support,^34,44,47,51,55,56^ foam,^32,35,39,42,45,46,54^ tandem stance^53^ and one-leg stance^32,39,42,43^) in combination with visual alterations (i.e. eyes closed,^32,34,35,39,42–47,51,54–56^ and moving visual scene^34,35,44,47,51,55,56^) was assessed in 15 studies. Meta-analysis of equilibrium scores revealed a significant improvement, with moderate heterogeneity. Results of equilibrium scores of studies that could not be pooled were in line with the meta-analyses.^34,51^

Meta-analysis of center of gravity sway velocity revealed a significant improvement when standing on a foam surface with eyes closed, but not for one-leg stance with eyes closed. Center of pressure sway area and sway velocity improved after repositioning maneuvers.^43,46,54^ In accordance with center of gravity sway velocity, performance time increased when standing on a foam surface with altered vision^35^ but not for tandem stance and one-leg stance with eyes closed.^53^

Postural control during vestibular alterations, combined with visual alterations (i.e. head movements and a moving visual scene) was compared between and controls in three studies.^24,26,54^ Results on sway area and velocity were conflicting.^26,54^ Performance time did not significantly differ.^24^

The impact of repositioning maneuvers on postural control during vestibular and visual alterations was assessed in two studies.^40,54^ Significant improvements in sway area and velocity were found.^40,54^

Postural control during visual, vestibular and alterations of base of support was compared between patients and controls in two studies.^17,24^ Patients presented increased accelerations^24^ and a decreased performance time.^17,24^

None of the included studies reported on the impact of repositioning maneuvers.

*Stability in gait* was compared between patients and controls in two studies*.*^18,23^ The root mean square of accelerations of the trunk, a measure for the amount of change in velocity, was compared in two studies.^18,23^ The root mean square of accelerations of head and trunk were generally decreased in patients, but significant differences were found only in rotatory movements of head and trunk, and in lateroflexion of the head.^18^ Results on flexion/extension movements of the trunk were conflicting.^18,23^

*Gait variability*, step/stride regularity and gait symmetry were compared between patients and controls in one study.^18^ Gait variability was increased in patients in flexion/extension and lateroflexion movements of the head, and in rotatory and lateroflexion movements of the trunk. The harmonic ratio, a measure of gait smoothness, was significantly decreased in patients in flexion/extension movements of the head, and in rotatory and flexion/extension movements of the trunk. Decreased consistency of gait was found in detected with a decreased step regularity of the head and a lower stride regularity in rotatory movements of the head. Patients also presented a reduced symmetry in flexion/extension movements of the trunk.

One study reported a significant improvement of coefficient of variations of stride time after repositioning maneuvers. Coefficient of variations of step width and stride length did, however, not improve.^52^

## Discussion

The aim of this study was to explore the impact of benign paroxysmal positional vertigo and repositioning maneuvers on the different components of postural control, with respect to the systems framework.^7^ Main findings are: 1) patients demonstrated a significantly altered perception of verticality and more postural sway during visual and multiple sensory alterations, but this recovered after repositioning maneuvers, 2) limits of stability significantly decreased in older patients but does not seem to improve after repositioning maneuvers, 3) although critical for fall avoidance,^58^ literature on biomechanical constraints, reactive postural control and transitions and anticipatory control was scarce. These findings imply that benign paroxysmal positional vertigo negatively affects several components of postural control. Except for one-leg stance, repositioning maneuvers significantly improve postural control. However, older adults may need additional rehabilitation to improve their limits of stability. These results may partly explain their increased odds of falling, and the improvement on falls and fear of falling after repositioning maneuvers.

Patients demonstrated an altered perception of verticality, which is important for establishing an efficient “starting position” for postural control and correlates, together with limits of stability, with fear of falling.^7^ However, only studies that used the subjective visual vertical to measure verticality were found, but more functional assessments (e.g. realignment of the trunk to the vertical) are recommended for future research. Limits of stability were only impaired in older patients. This may be caused by an increased fear of falling or altered biomechanical constraints experienced by older, but not by younger patients.^59^ Since older adults often already experience age-related changes in postural control, they could experience a higher impact of benign paroxysmal positional vertigo on postural control compared to younger ones. The limits of stability of older patients also does not seem to improve after treatment, indicating that they may need additional rehabilitation after repositioning maneuvers.

Persistent decreased postural control while diagnostic tests for benign paroxysmal positional vertigo are negative, may also be due to the presence of coexisting vestibular disorders, which is highly prevalent in patients with benign paroxysmal positioning vertigo,^60^ and/or persistent postural perceptual dizziness.^61^

On sensory orientation, patients were still able to reweight the sensory input from the visual (and vestibular) system to reduce their postural sway when the surface was altered during bipedal stance. However, when visual information was altered, postural sway increased significantly. The reweighting of the somatosensory (and vestibular) input was insufficient under this condition, but this improved after repositioning maneuvers.

Despite an increased postural sway, patients were still able to maintain a standing position for 30 s when visual input was altered. Performance time only significantly decreased both visual and somatosensory input was altered. This was also reflected in their decreased vestibular ratio, which improved after repositioning maneuvers, suggesting and improved reliance on vestibular information post-treatment. These results imply that, when measuring postural sway, altering visual input is a sensitive measure for assessing the impact of benign paroxysmal positional vertigo on postural control and the effectiveness of repositioning maneuvers. However, when measuring performance time, task difficulty should be increased by simultaneously altering visual and somatosensory inputs. When the base of support was reduced (as in one-leg stance and tandem stance), patients experienced more difficulties than controls, but this did not improve after repositioning maneuvers. Patients possibly need additional rehabilitation to recover one-leg and tandem stance, as indicated in randomized control trials where only additional rehabilitation after repositioning maneuvers led to significant improvements in one-leg stance.^32,39,43^

Treatment with repositioning maneuvers significantly reduced the time to complete the timed chair stand test (from 19.63 to 13.61 s). Since performance is influenced by factors beyond muscle strength^62^ (physical fitness, postural sway, anxiety^63^) and improvements were established one week post-treatment, the results likely stem from these other factors. Nevertheless, the time reduction exceeded the minimal clinically important difference of 2.3 s^64^ suggesting a meaningful contribution to the reduced number of falls after repositioning maneuvers.^5^

There were some limitations to this study. As only 9 studies performed vestibular function tests to exclude patients with coexisting vestibular disorders, this study includes patients with and without a coexisting vestibular disorder. A coexisting vestibular disorder can interfere with postural control, the treatment effect of repositioning maneuvers and consequently the results of this study. Overall, heterogeneity within and between the included studies (timing of first treatment, duration of complaints, number of repositioning maneuvers) was large. Also, age ranges within included studies were broad. We could only differentiate between age groups where data permitted, despite known age-related declines in postural control performance.^65,66^ Additionally, small sample sizes and varied assessment methods limited meta-analysis and complicated interpretation.

Nevertheless, this is the first systematic review that provides an overview of impairments of postural control and the impact of repositioning maneuvers in people with benign paroxysmal positional vertigo. Study selection was performed by 2 independent researchers in 3 electronic databases and reference lists, in order to include all relevant articles. The assessment of internal validity and risk of bias resulted in the exclusion of 5 articles. By using the systems framework and by including both clinical and kinematic data, we provide a broad overview of the different underlying components of postural control and the degree to which they have been investigated in the existing literature.

In summary, benign paroxysmal positional vertigo significantly affects the perception of verticality and sensory orientation with visual and multiple alterations and stability in gait. In older patients, limits of stability is also impaired. Except for one-leg stance, and limits of stability in older adults, repositioning maneuvers are able to significantly improve the underlying components of postural control. Our findings on decreased postural control may partly explain the increased odds of falling in people with benign paroxysmal positional vertigo. This emphasizes the importance for screening and treating benign paroxysmal positional vertigo in people with decreased postural control, especially when experiencing dizziness for <1 min and when symptoms are triggered by rolling in bed. In older adults, however, greater awareness for benign paroxysmal positional vertigo is indicated, since they often present with less-classic symptoms, which increases their odds of falling.

More research is required for conclusive results, but screening and treating benign paroxysmal positional vertigo with repositioning maneuvers can already lead to improvements in postural control and fall prevention.

**Clinical messages**

- Benign Paroxysmal Positional Vertigo is a vestibular disorder that affects several domains of postural control, such as the perception of verticality, sensory orientation and stability in gait.
- Treatment with repositioning maneuvers improves postural control on these domains, except for limits of stability in older patients, and one-leg stance and tandem stance.
- When measuring postural sway, altering visual input during bipedal stance is a sensitive measure for assessing the impact of benign paroxysmal positional vertigo on postural control and the effectiveness of repositioning maneuvers. However, when measuring performance time, task difficulty should be increased by simultaneously altering visual and somatosensory inputs.

## Supplemental Material

sj-docx-1-cre-10.1177_02692155241292662 - Supplemental material for Several components of postural control are affected by benign paroxysmal positional vertigo but improve after particle-repositioning maneuvers: A systematic review and meta-analysis Supplemental material, sj-docx-1-cre-10.1177_02692155241292662 for Several components of postural control are affected by benign paroxysmal positional vertigo but improve after particle-repositioning maneuvers: A systematic review and meta-analysis by Sara Pauwels, Laura Casters, Pieter Meyns, Nele Lemkens, Winde Lemmens, Kenneth Meijer, Raymond van de Berg and Joke Spildooren in Clinical Rehabilitation
