# Supplementary material for: Several components of postural control are affected by benign paroxysmal positional vertigo but improve after particle-repositioning maneuvers: A systematic review and meta-analysis
Source: Clin Rehabil. 2024 Nov 5;39(1):3–22. doi: 10.1177/02692155241292662 (PMC11776354; doi:10.1177/02692155241292662)
Supplement: sj-docx-1-cre-10.1177_02692155241292662 - Supplemental material for Several components of postural control are affected by benign paroxysmal positional vertigo but improve after particle-repositioning maneuvers: A systematic review and meta-analysis [file sj-docx-1-cre-10.1177_02692155241292662.docx]

**Supplementary materials
Supplementary materials 1 - Search string**

*Pubmed 493*

("Benign Paroxysmal Positional Vertigo"[MeSH Terms] OR "Benign Paroxysmal Positional Vertigo"[Title/Abstract] OR "Benign Paroxysmal Positioning Vertigo" [Title/Abstract] OR "BPPV"[Title/Abstract]) AND ("postural balance"[MeSH Terms] OR "postural balance"[Title/Abstract] OR "Balance"[Title/Abstract] OR "postural stability"[Title/Abstract] OR "Gait"[MeSH Terms] OR "Gait"[Title/Abstract] OR "gait analyses"[Title/Abstract] OR "postur*"[Title/Abstract] OR "Posturography"[Title/Abstract] OR "Walking"[MeSH Terms] OR "walk*"[Title/Abstract] OR "Locomotion"[Title/Abstract] OR "gait stability"[Title/Abstract])

*Web of Science 333*

("Benign Paroxysmal Positioning Vertigo" OR "Benign Paroxysmal Positional Vertigo" OR "BPPV") (Topic) and ("Balance" OR "Postural Balance" OR "Stability" OR "Postural Stability" OR "Posturography" OR "Gait" OR "Walking" OR "Gait Stability" OR "Gait analyses") (Topic)

*Scopus 686*

( TITLE-ABS-KEY ( ( "Benign Paroxysmal Positioning Vertigo" OR "Benign Paroxysmal Positional Vertigo"   OR  "BPPV" ) )  AND  TITLE-ABS-KEY ( ( "Balance"  OR  "Postural Balance"  OR  "Stability"  OR  "Postural Stability"  OR  "Posturography"  OR  "Gait"  OR  "Walking"  OR  "Gait Stability"  OR  "Gait analyses" ) ) )

| Supplementary materials 2 - Components of postural control and applied methodology in the included studies | | |
| --- | --- | --- |
| Components of postural control | **Applied tests/tasks in the included studies** | **Applied outcome variables in the included studies** |
| Biomechanical constraints | Lower limb test(35) | Seconds(35) |
| Verticality | Subjective visual vertical(28–31,37,38,48) | Deviation (°)(28–30,37,38,48); n (%) abnormal deviation (≥ 3°)(31) |
| Limits of stability | Limits of stability Area - Balance Rehabilitation Unit (BRU^TM^)(26,40,54) Limits of stability - NeuroCom(39,42) Computerised Dynamic Posturography(50) | LoS area cm^2 ;^ (26,40,54) Movement Velocity (°/s)(39,42,50) , Endpoint Excursion (%LoS)(42,50), Maximum Excursion (%LoS)(39,42,50), Directional Control(%LoS)(42,50) |
| Transitions and anticipatory postural adjustment | Shortened Functional Mobility test(23) | Number of obstacles touched(23), time (s)(23) |
| Reactive Postural responses | Motor Control test- Neurocom(56) | Strength score(56) |
| Sensory Orientation | Sensory Organization Test - Neurocom(34,36,44,47,51,55,56)  firm surface, eyes open(16,24–26,33,41,45–47,54,56)  firm surface, eyes closed(15,16,22,24,26,31,33,35,39–42,45,46,54).  firm surface, moving visual scene(26,35,40,54)  Firm surface, head movements(24) Foam surface, eyes open(24,25,32,35,39,40,42,43,45,46)  Foam surface, eyes closed(17,24–26,45,46,54)  Foam surface, moving visual scene(35)  Tandem stance(22,25)  One leg stance(22,32,33,39,42)  One leg stance, eyes closed(32,39,42,43)  Tandem stand, eyes closed(22,25)  Head movements, moving visual scene (26,40,54) Head movements, eyes closed(24)  Foam surface, eyes closed, head movements(17,24) | Equilibrium score(34,44,47,51,55,56); Composite Score(36,47,56); Sensory ratio(36,46,47,50) COG sway velocity (°/s)(32,39,42,45)  COP sway area (cm^2^)(26,40,54); COP sway velocity (cm/s)(26,40,54); COP sway velocity AP & ML (cm/s)(33,41); COP displacement (% stillness)(43); COP path length (mm)(25);  Range AP (cm/s^2^)(25), Range ML (cm/s^2^)(25), Peak velocity AP (cm/s)(25), Peak velocity ML (cm/s)(25), RMS AP (25), RMS (25)  COP displacement (%frontal plane stability)(43)  Time(s)(17,22,24,31,35,53) |
| Stability in gait | Level walking(18,23,52) | RMS acceleration, harmonic ratio, gait symmetry & variability, step & stride regularity head and trunk along AP, ML and vertical axis(18)  CV of stride time (%)(52), CV op step width (%)(52), CV of stride length (%)(52)  RMS TAX, TAY, TAR, TRV, TPV, TYV(23) |

*Abbreviations: N, number of participants; LoS, limits of stability; AP, antero-posterior; ML, medio-lateral; COG, center of gravity; COP, center of pressure; CoM, center of mass; RMS, root mean square; CV, coefficient of variation; TAX, acceleration along the fore-aft axis; TAY, acceleration along the medio-lateral axis for the trunk; TAR, resultant acceleration; TRV, angular velocity about the roll axis; TPV, angular velocity about the pitch axis; TYV, angular velocity about the yaw axis*

**Supplementary materials 3 – Risk-of-bias assessment**

**3a. Risk-of-bias assessment for case-control studies**

| Author | Q1 | Q2 | Q3 | Q4 | Q5 | Q6 | Q7 | Q8 | Q9 | Q10 | Risk of bias |
| --- | --- | --- | --- | --- | --- | --- | --- | --- | --- | --- | --- |
| Agarwal et al., 2012 | Y | N | Y | N | Y | Y | Y | Y | Y | Y | Low |
| Best et al. 2006 | N | N | Y | Y | Y | Y | Y | Y | N | Y | Low |
| Chang et al. 2006 | N | N | N | Y | N | N | N | N | N | N | High |
| Cohen et al., 2011 | Y | N | Y | N | N | N | N | Y | Y | Y | Moderate |
| Cohen et al., 2014 | N | N | Y | N | Y | Y | Y | Y | Y | Y | Low |
| Cohen and Sangi-Haghpeykar 2012 | N | N | N | Y | N | Y | Y | Y | Y | Y | Moderate |
| D’Silva et al., 2017 | Y | Y | Y | N | N | Y | Y | Y | Y | Y | Low |
| Kollén et al., 2012 | N | N | Y | N | Y | Y | Y | Y | N | Y | Moderate |
| Lindell et al., 2021 | Y | N | Y | Y | Y | N | N | Y | Y | Y | Low |
| Lin et al., 2020 | N | N | N | N | N | Y | N | Y | N | Y | High |
| Monteiro et al., 2012 | Y | Y | Y | N | Y | Y | Y | Y | Y | Y | Low |
| Mulavara et al., 2013 | N | N | Y | N | Y | Y | Y | Y | Y | Y | Low |
| Mutlu and Topcu 2022 | N | N | N | N | N | N | N | Y | N | Y | High |
| Nair et al., 2018 | N | N | Y | N | Y | N | N | Y | N | Y | High |
| Hong et al. 2008 | Y | Y | N | N | N | N | N | Y | N | Y | High |
| Lee et al. 2014 | Y | Y | Y | Y | Y | N | N | Y | N | Y | Low |
| Zhang et al., 2021 | Y | Y | N | N | N | Y | Y | Y | N | Y | Moderate |

*Abbreviations: Y: “yes”; N: “no” ; U: “Unclear”; Q1: Were the groups comparable other than the presence of the disease in cases or absence of disease in controls?; Q2: Were cases and controls matched appropriately?; Q3: Were the same criteria used for identification of cases and controls?; Q4: Was exposure measured in a standard, valid and reliable way?; Q5: Was exposure measured in the same way for cases and controls?; Q6: Were confounding factors identified?; Q7: Were strategies to deal with confounding factors stated?; Q8: Were outcomes assessed in a standard, valid and reliable way for cases and controls?; Q9: Was the exposure period of interest long enough to be meaningful?;*

**3b. Risk-of-bias assessment for quasi-experimental studies**

| Author | Q1 | Q2 | Q3 | Q4 | Q5 | Q6 | Q7 | Q8 | Q9 | Risk of bias |
| --- | --- | --- | --- | --- | --- | --- | --- | --- | --- | --- |
| Abou-Elew et al., 2011 | Y | Y | NA | N | Y | Y | NA | N | N | Moderate |
| Assal et al. 2022 | Y | Y | Y | Y | Y | Y | Y | Y | N | Low |
| Blatt et al., 2000 | Y | Y | NA | N | Y | Y | NA | N | N | Moderate |
| Bulğurcu et al., 2021 | Y | Y | N | Y | Y | Y | Y | Y | Y | Low |
| Çelebisoy et al., 2009 | Y | N | Y | Y | Y | Y | Y | N | Y | Low |
| Chang et al., 2008 | Y | Y | Y | Y | Y | Y | Y | Y | N | Low |
| Cohen & Kimball, 2004 | Y | N | Y | Y | Y | Y | Y | N | N | Low |
| Cohen & Sangi-Haghpeykar, 2010 | Y | Y | Y | Y | Y | Y | Y | Y | Y | Low |
| Cohen-Shwartz et al., 2020 | Y | Y | Y | Y | Y | Y | Y | Y | Y | Low |
| Di Girolamo et al., 1998 | Y | Y | N | Y | Y | Y | Y | N | N | Low |
| Di Girolamo et al., 2000 | Y | N | N | Y | Y | Y | N | N | N | High |
| Faralli et al. 2016 | Y | Y | Y | y | y | y | y | N | Y | Low |
| Kasse et al., 2012 | Y | y | Y | Y | Y | Y | Y | N | N | Low |
| Kollén et al., 2006 | Y | Y | NA | N | Y | Y | NA | N | N | Moderate |
| Lança et al., 2013 | Y | Y | NA | N | Y | Y | NA | N | N | Moderate |
| Lim et al., 2021 | Y | Y | NA | N | Y | Y | NA | N | N | Moderate |
| Navarro et al. 2023 | Y | N | Y | Y | N | Y | Y | Y | N | Moderate |
| Omara et al., 2017 | Y | Y | N | Y | Y | Y | Y | N | N | Low |
| Ribeiro et al., 2017 | Y | Y | Y | Y | Y | Y | Y | Y | N | Low |
| Silva et al., 2014 | Y | Y | NA | N | Y | Y | NA | N | N | Moderate |
| Stambolieva & Angov, 2010 | Y | N | Y | Y | Y | Y | Y | N | N | Low |
| Stambolieva & Angov, 2006 | Y | N | Y | Y | Y | Y | Y | N | N | Low |
| Vaz et al., 2013 | Y | Y | NA | N | Y | Y | NA | N | N | Moderate |
| Faralli et al. 2011 | Y | N | Y | Y | Y | Y | Y | N | N | Moderate |
| Ferreira, Ganança, and Caovilla 2017 | Y | Y | NA | N | Y | Y | NA | N | N | Moderate |
| Taçalan et al. 2021 | Y | Y | Y | Y | Y | Y | Y | Y | Y | Low |

*Abbreviations: Y: “yes”, N: ”No”, NA: “not applicable”; Q1: Is it clear in the study what is the ‘cause’ and what is the ‘effect’?; Q2: Were the participants included in any comparison similar?; Q3: Were the participants included in any comparisons receiving similar treatment/care, other than the exposure or intervention of interest
Q4: Was there a control group?; Q5: Were there multiple measurements of the outcome both pre and post the intervention/ exposure?; Q6: Was follow-up complete, and if not, were differences between groups in terms of their follow up adequately described and analyzed?; Q7: Were the outcomes of participants included in any comparison measured in the same way?; Q8: Were outcomes measured in a reliable way?; Q9: Was appropriate statistical analysis used?* **Supplementary materials 4 – Meta-analyses on verticality**

**4a. Meta-analysis on the impact of BPPV on subjective visual vertical**A comparison of the subjective visual vertical (degrees) of people with BPPV (pwBPPV) versus control.**
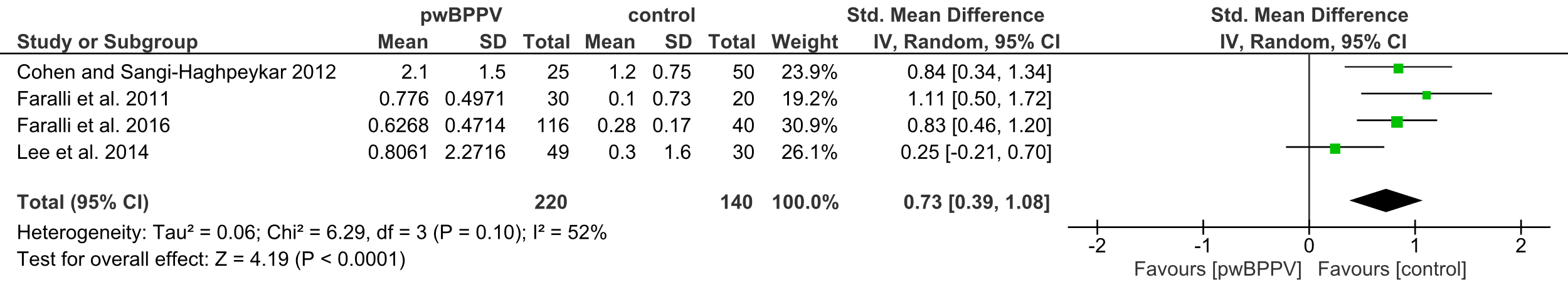
**

*Legend: A significant result is visualized by the diamond shape not crossing the central vertical line.
Abbreviations: pwBPPV, people with BPPV; Std, Standardized; IV, Inversed Variance; CI, confidence interval.*

**4b. Meta-analysis on treatment effect of particle-repositioning maneuvers (PRM) on subjective visual vertical**A comparison of the subjective visual vertical (degrees) of people with BPPV before treatment with PRM (Pre) and after treatment with PRM (Post).


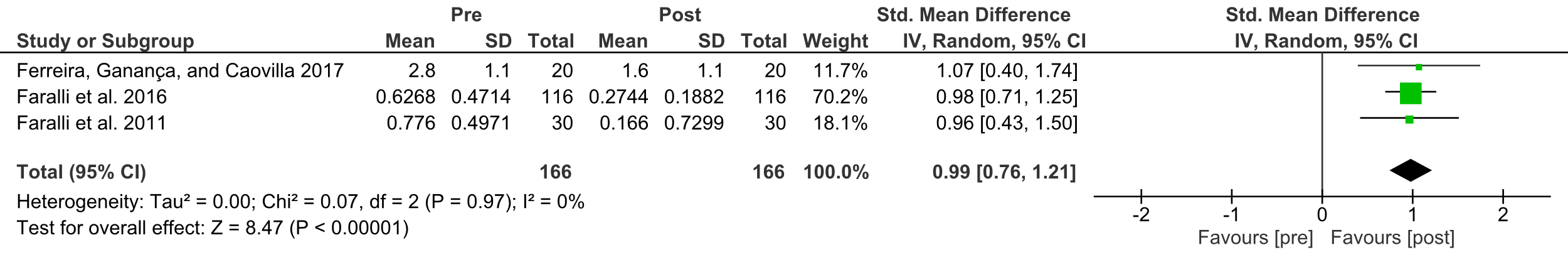


*Legend: A significant result is visualized by the diamond shape not crossing the central vertical line.
Abbreviations: Std, Standardized; IV, Inversed Variance; CI, confidence interval; PRM, particle-repositioning maneuvers.*

**Supplementary materials 5 – Meta-analysis of the impact of BPPV and treatment effect of PRM on sensory orientation: composite score**

**5a. Meta-analysis on impact of BPPV on composite score**A comparison of the composite score of people with BPPV (pwBPPV) versus control
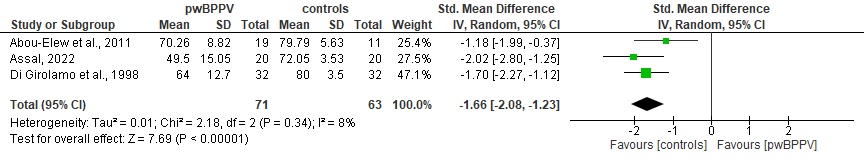
*Legend: A significant result is visualized by the diamond shape not crossing the central vertical line.
Abbreviations: Std, Standardized; IV, Inversed Variance; CI, confidence interval.*

**5b. Meta-analysis on treatment effect of particle repositioning maneuvers (PRM) on composite score**
A comparison of composite scores of people with BPPV before treatment with PRM (Pre) and after treatment with PRM (Post).
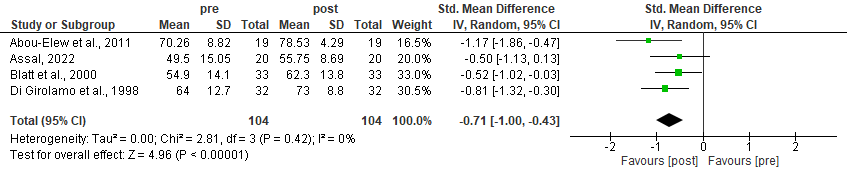


*Legend: A significant result is visualized by the diamond shape not crossing the central vertical line.
Abbreviations: Std, Standardized; IV, Inversed Variance; CI, confidence interval; PRM, particle repositioning maneuvers.*

**Supplementary materials 6 – Meta-analyses of treatment effect of particle-repositioning maneuvers (PRM) on sensory orientation: sensory ratios**

**6a. Meta-analysis on treatment effect of PRM on vestibular ratio**A comparison of vestibular ratio of people with BPPV before treatment with PRM (Pre) and after treatment with PRM (Post)

*
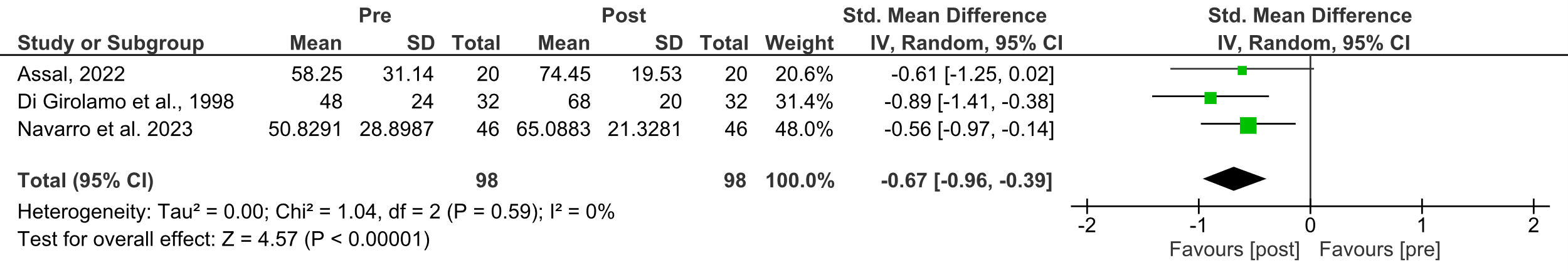
Legend: A significant result is visualized by the diamond shape not crossing the central vertical line.
Abbreviations: Std, Standardized; IV, Inversed Variance; CI, confidence interval; PRM, particle-repositioning maneuvers.*

**6b. Meta-analysis on treatment effect of PRM on visual ratio**A comparison of visual ratio of people with BPPV before treatment with PRM (Pre) and after treatment with PRM (Post)


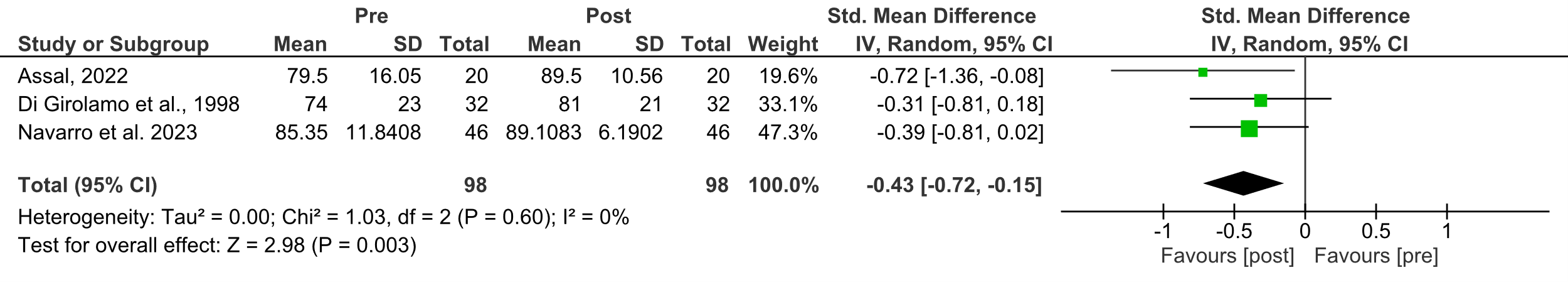


*Legend: A significant result is visualized by the diamond shape not crossing the central vertical line.
Abbreviations: Std, Standardized; IV, Inversed Variance; CI, confidence interval; PRM, particle-repositioning maneuvers.*

**6c. Meta-analysis on treatment effect of PRM on somatosensory ratio**A comparison of somatosensory ratio of people with BPPV before treatment with PRM (Pre) and after treatment with PRM (Post)


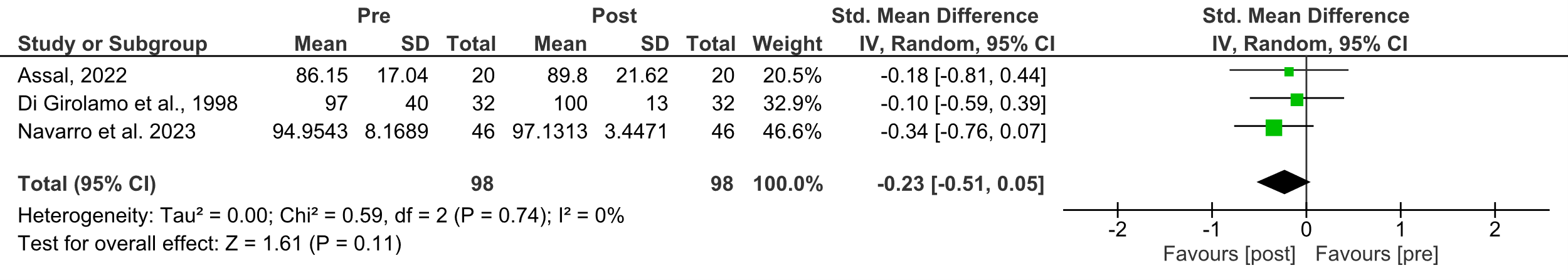


*Legend: A non-significant result is visualized by the diamond shape crossing the central vertical line.
Abbreviations: Std, Standardized; IV, Inversed Variance; CI, confidence interval; PRM, particle-repositioning maneuvers.*

**Supplementary materials 7 –** **Meta-analyses of treatment effect of particle-repositioning maneuvers (PRM) on sensory orientation: postural control without sensory alterations**

**7a. Meta-analysis on treatment effect of PRM on equilibrium score**A comparison of equilibrium scores of people with BPPV before treatment with PRM (Pre) and after treatment with PRM (Post)

**
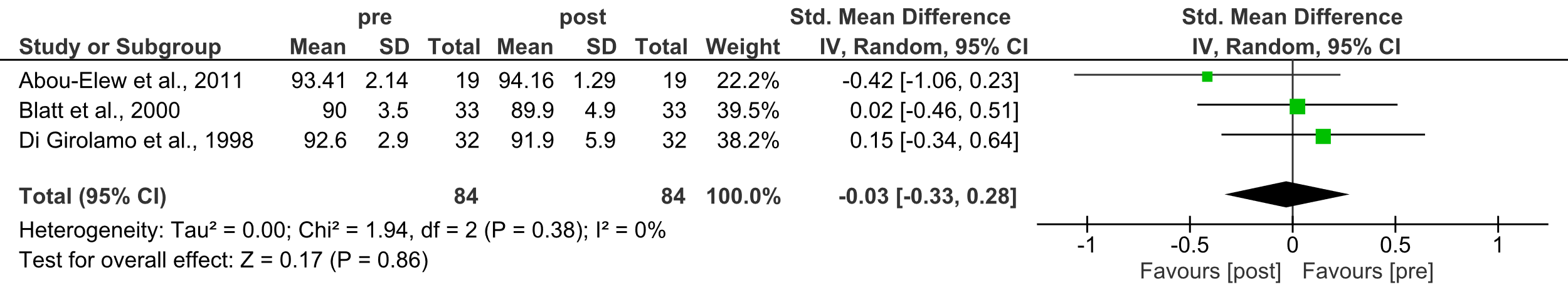
***Legend: A non-significant result is visualized by the diamond shape crossing the central vertical line.
Abbreviations: Std, Standardized; IV, Inversed Variance; CI, confidence interval; PRM, particle-repositioning maneuvers.*

**7b. Meta-analysis on treatment effect of PRM on sway velocity**A comparison of center of gravity sway velocity (°/s) on postural control without sensory alterations of people with BPPV before treatment with PRM (Pre) and after treatment with PRM (Post)


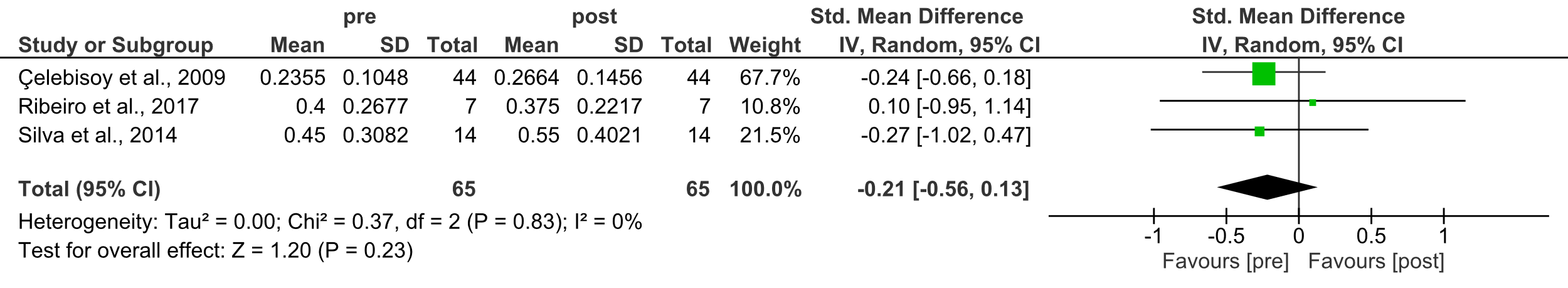


*Legend: A non-significant result is visualized by the diamond shape crossing the central vertical line.
Abbreviations: Std, Standardized; IV, Inversed Variance; CI, confidence interval; PRM, particle-repositioning maneuvers.*

**Supplementary materials 8 –** **Meta-analyses of treatment effect of particle-repositioning maneuvers (PRM) on sensory orientation: postural control with visual alterations**

**8a. Meta-analysis on treatment effect of PRM on equilibrium scores during eyes closed**A comparison of equilibrium scores during eyes closed of people with BPPV before treatment with PRM (Pre) and after treatment with PRM (Post)

**
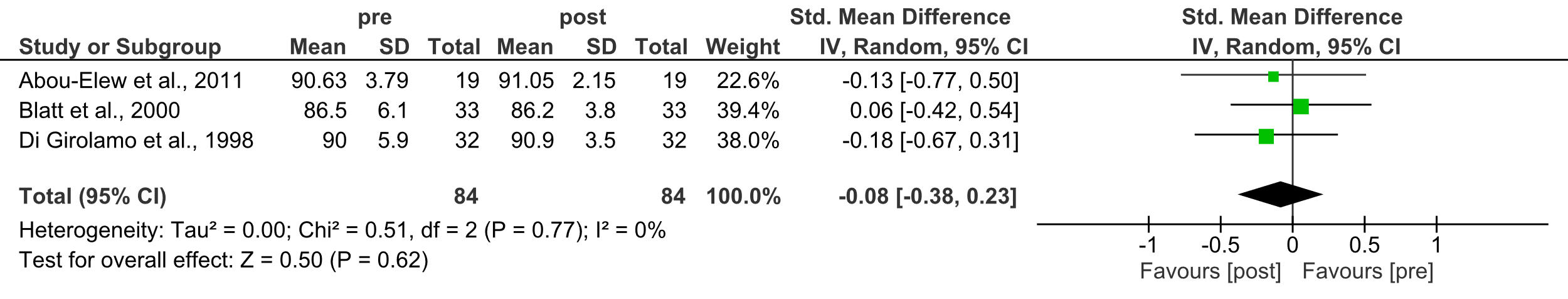
**

*Legend: A non-significant result is visualized by the diamond shape crossing the central vertical line.
Abbreviations: Std, Standardized; IV, Inversed Variance; CI, confidence interval; PRM, particle-repositioning maneuvers.*

**8b. Meta-analysis on treatment effect of PRM on sway velocity**A comparison of center of gravity sway velocity (°/s) during eyes closed of people with BPPV before treatment with PRM (Pre) and after treatment with PRM (Post)


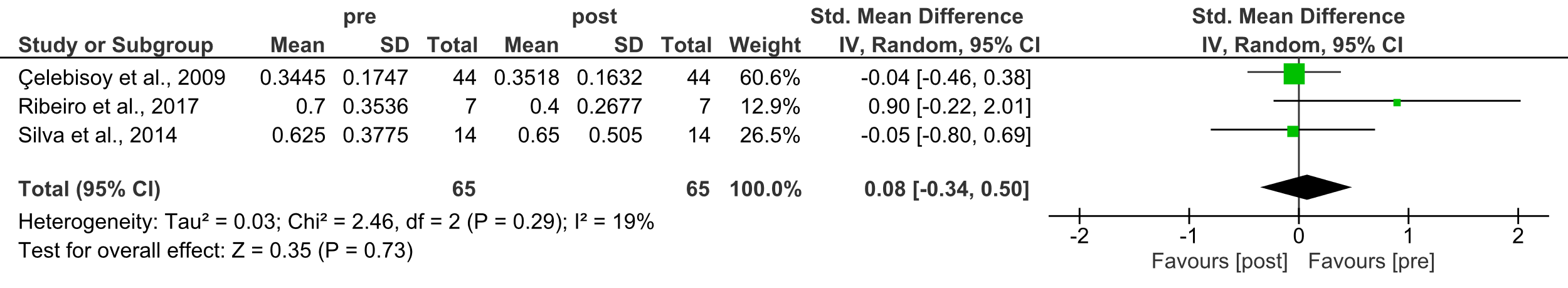


*Legend: A non-significant result is visualized by the diamond shape crossing the central vertical line.
Abbreviations: Std, Standardized; IV, Inversed Variance; CI, confidence interval; PRM, particle-repositioning maneuvers.*

**8c. Meta-analysis on treatment effect of PRM on equilibrium scores during altered visual information**A comparison of equilibrium scores during altered visual information of people with BPPV before treatment with PRM (Pre) and after treatment with PRM (Post)

**
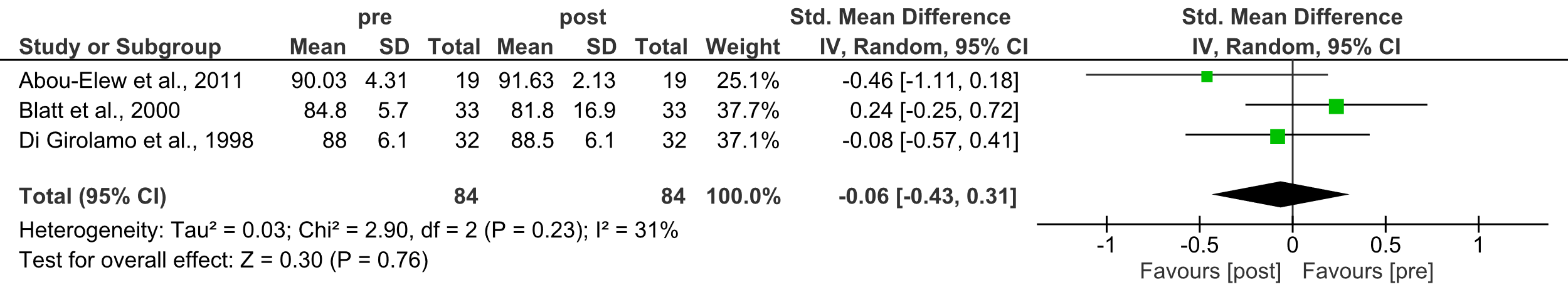
**

*Legend: A non-significant result is visualized by the diamond shape crossing the central vertical line.
Abbreviations: Std, Standardized; IV, Inversed Variance; CI, confidence interval; PRM, particle-repositioning maneuvers.*

**Supplementary materials 9 –** **Meta-analyses of treatment effect of particle-repositioning maneuvers (PRM) on sensory orientation: postural control with alterations of the base of support**

**9a. Meta-analysis on treatment effect of PRM on equilibrium scores during sway referenced support**A comparison of equilibrium scores during sway referenced support of people with BPPV before treatment with PRM (Pre) and after treatment with PRM (Post)

**
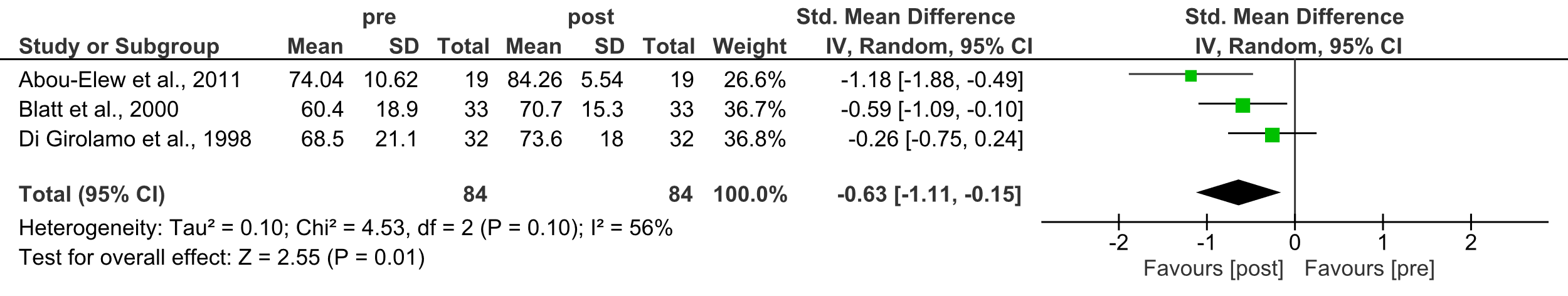
**

*Legend: A significant result is visualized by the diamond shape not crossing the central vertical line.
Abbreviations: Std, Standardized; IV, Inversed Variance; CI, confidence interval; PRM, particle-repositioning maneuvers.*

**9b. Meta-analysis on treatment effect of PRM on sway velocity during foam**A comparison of center of gravity sway velocity (°/s) during standing of a foam of people with BPPV before treatment with PRM (Pre) and after treatment with PRM (Post)


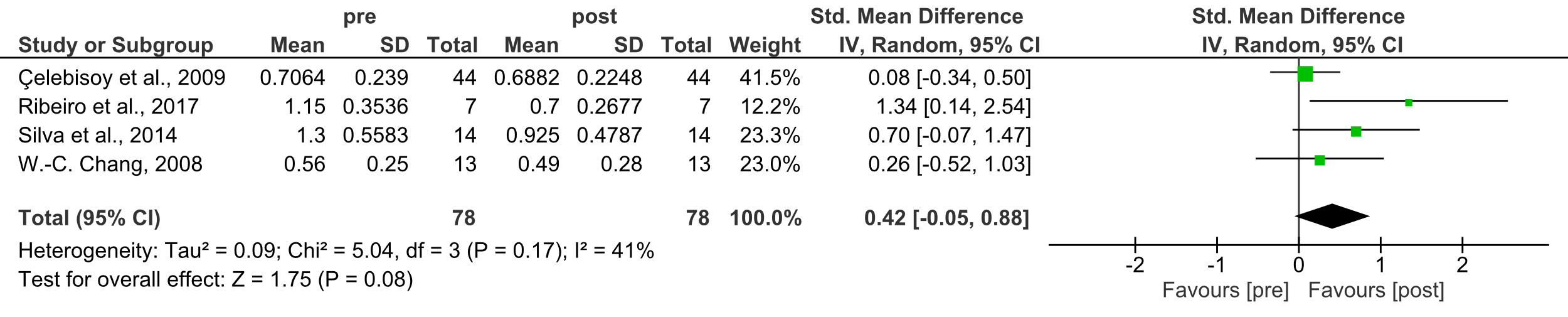


*Legend: A non-significant result is visualized by the diamond shape crossing the central vertical line.
Abbreviations: Std, Standardized; IV, Inversed Variance; CI, confidence interval; PRM, particle-repositioning maneuvers.*

**9c. Meta-analysis on treatment effect of PRM on sway velocity during one leg stanceµ**

A comparison of of center of gravity sway velocity (°/s) during one leg stance of people with BPPV before treatment with PRM (Pre) and after treatment with PRM (Post)

**
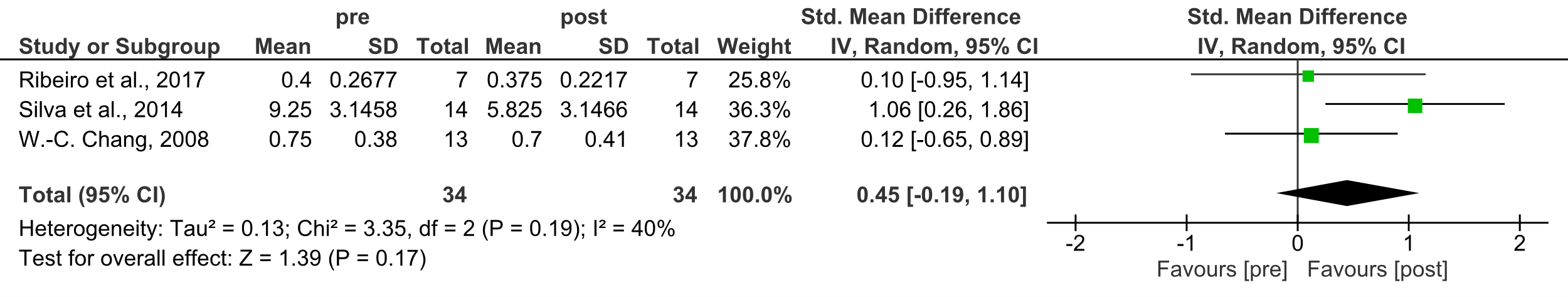
**

*Legend: A non-significant result is visualized by the diamond shape crossing the central vertical line.
Abbreviations: Std, Standardized; IV, Inversed Variance; CI, confidence interval; PRM, particle-repositioning maneuvers.*

**Supplementary materials 10 –** **Meta-analyses of treatment effect of particle-repositioning maneuvers (PRM) on sensory orientation: postural control with more than one sensory alteration**

**10a. Meta-analysis on treatment effect of PRM on equilibrium scores during sway-referenced support surface with eyes closed**

A comparison of equilibrium scores during sway-referenced support surface with eyes closed of people with BPPV before treatment with PRM (Pre) and after treatment with PRM (Post)

**
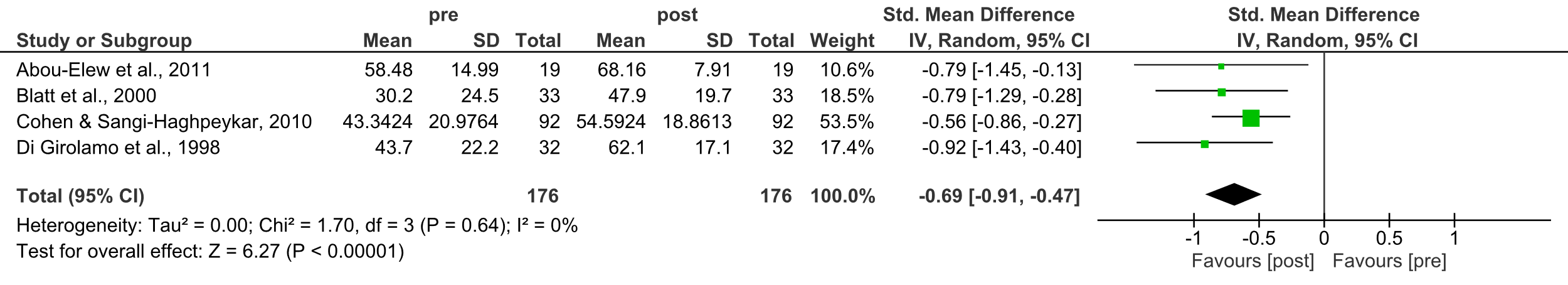
**

*Legend: A significant result is visualized by the diamond shape not crossing the central vertical line.
Abbreviations: Std, Standardized; IV, Inversed Variance; CI, confidence interval; PRM, particle-repositioning maneuvers.*

**10b. Meta-analysis on treatment effect of PRM on equilibrium scores during sway-referenced support surface and visual surround**

A comparison of equilibrium scores during sway-referenced support surface and visual surround of people with BPPV before treatment with PRM (Pre) and after treatment with PRM (Post) **
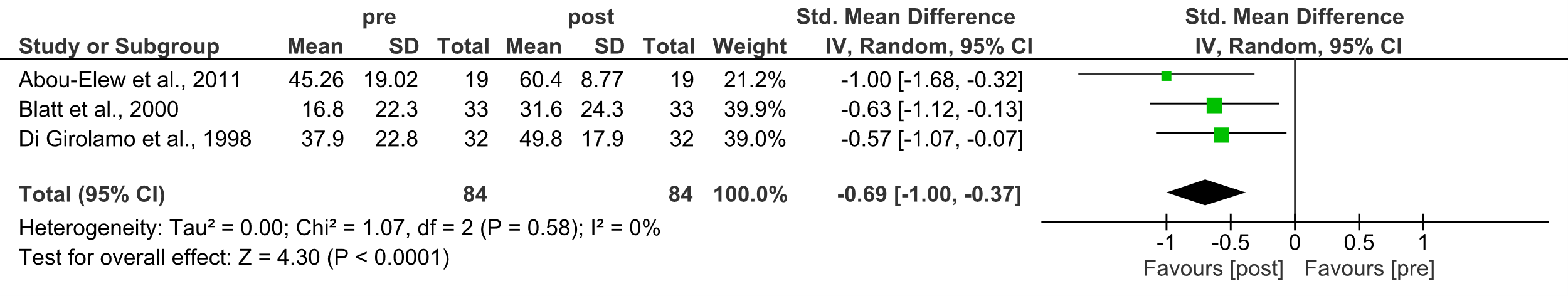
**

*Legend: A significant result is visualized by the diamond shape not crossing the central vertical line.
Abbreviations: Std, Standardized; IV, Inversed Variance; CI, confidence interval; PRM, particle-repositioning maneuvers.*

**10c. Meta-analysis on treatment effect of PRM on sway velocity during standing on a foam surface with eyes closed**

A comparison of center of gravity sway velocity (°/s) during standing on a foam surface with eyes closed of people with BPPV before treatment with PRM (Pre) and after treatment with PRM (Post)**
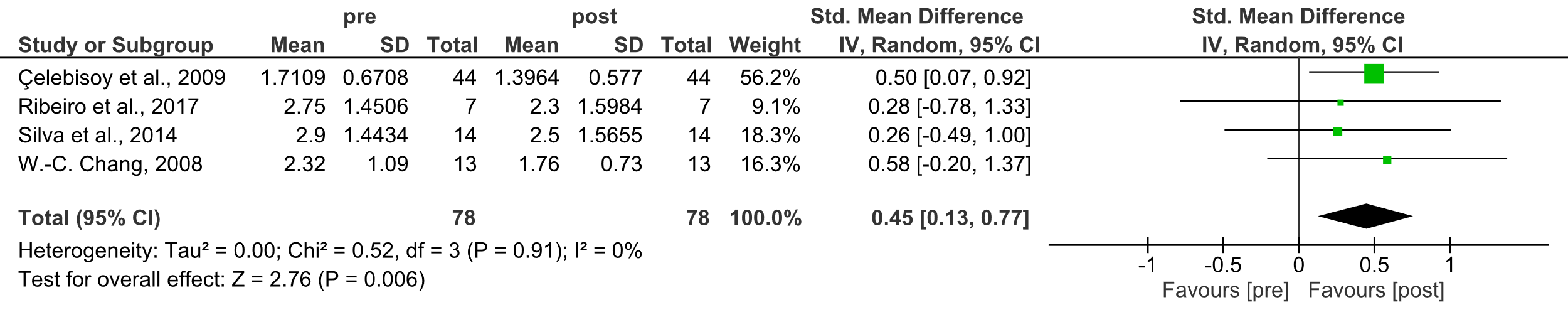
**

*Legend: A significant result is visualized by the diamond shape not crossing the central vertical line.
Abbreviations: Std, Standardized; IV, Inversed Variance; CI, confidence interval; PRM, particle-repositioning maneuvers.*

**10d. Meta-analysis on treatment effect of PRM on sway velocity during one-leg stance with eyes closed**

A comparison of center of gravity sway velocity (°/s) during one-leg stance with eyes closed of people with BPPV before treatment with PRM (Pre) and after treatment with PRM (Post)

**
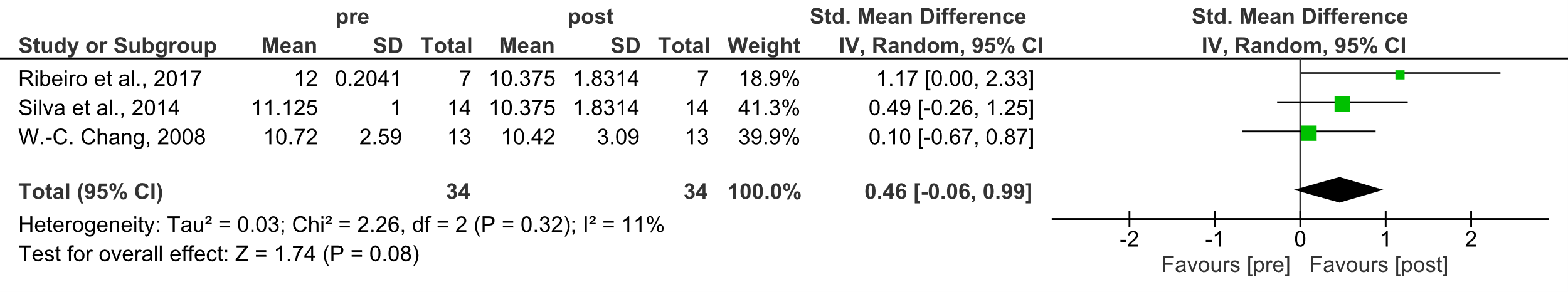
**

*Legend: A non-significant result is visualized by the diamond shape crossing the central vertical line.
Abbreviations: Std, Standardized; IV, Inversed Variance; CI, confidence interval; PRM, particle-repositioning maneuver*
